# Composite disturbance observer-based sliding mode control strategies for unmanned surface vehicles with unmatched disturbances

**DOI:** 10.1016/j.isci.2026.115320

**Published:** 2026-03-12

**Authors:** Yixin Su, Chenglong Gong, Zhengying Li, Danhong Zhang

**Affiliations:** 1 School of Automation, Wuhan University of Technology, Wuhan 430070, China; 2 School of Information Engineering, Wuhan University of Technology, Wuhan 430070, China

**Keywords:** Applied sciences, Engineering

## Abstract

Unmatched disturbances pose significant challenges to the control performance of unmanned surface vehicles (USVs), necessitating effective mitigation control strategies. This study proposes composite disturbance observer-based sliding mode control (SMC) strategies to address the trajectory tracking control problem of USVs under the influence of unmatched and matched disturbances, and input saturation. First, a composite disturbance observer is designed to estimate and compensate for the effects of both unmatched and matched disturbances. Subsequently, an SMC strategy is proposed to eliminate input discrepancies and ensure system state convergence within a designated region. Moreover, to mitigate the reliance on prior knowledge of the upper bounds of lumped disturbances, an improved SMC strategy is further proposed that incorporates a positive semi-definite barrier (PSDB) function. This strategy enhances the steady-state performance of the system and prevents excessive estimation of control gains. Finally, numerical simulations validate the effectiveness of the proposed control strategies.

## Highlights

• A disturbance observer is developed to estimate both matched and unmatched disturbances
• A composite disturbance observer-based SMC strategy is proposed
• Successfully addressed the input saturation problem for unmanned surface vehicles
• An improved SMC strategy with a positive semi-definite barrier function is proposed

## Graphical abstract

**Figure:**
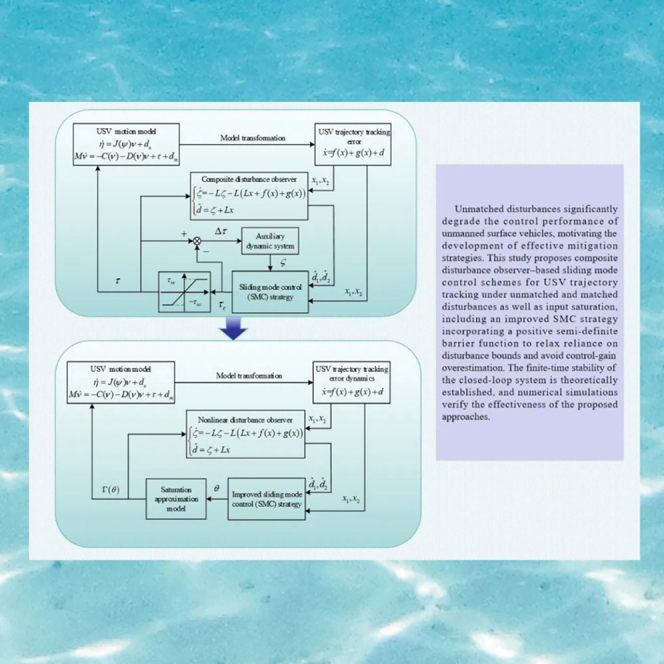

## Introduction

Unmanned surface vehicles (USVs) are intelligent robotic platforms capable of autonomous navigation or remote control. With high flexibility, reliability, and endurance, USVs have become essential tools for target tracking, security patrols, and resource exploration.^1^ Trajectory tracking is a fundamental function for the USV, and numerous advanced control strategies have been proposed to achieve this objective.^2^^,^^3^^,^^4^^,^^5^ However, trajectory tracking control strategies for USVs continue to face challenges from environmental disturbances, input saturation, and steady-state performance requirements. In particular, research on eliminating adverse effects of unmatched disturbances on the USV has yet to receive adequate attention.^6^

During operation, USVs are inevitably subjected to disturbances arising from wind, waves, and currents. Wang et al. employed a disturbance observer to address external disturbances and quantify system states, proposing a dynamic surface-based adaptive trajectory tracking control strategy to ensure bounded system errors.^7^ Liu et al. introduced an indirect adaptive disturbance observer to handle external disturbance amplitudes and developed a reconfigurable path-tracking control method, enabling the USV under multiple constraints to follow desired paths with controlled deviations.^8^ Deng et al. constructed a neural adaptive containment control framework with a finite-time disturbance observer to ensure that all followers in a USV formation avoid collisions while remaining within the convex hull spanned by leaders.^9^ To address lumped disturbances arising from actuator faults and external disturbances, Zhang et al. integrated a dynamic event-triggering mechanism to design an anti-disturbance control strategy, thereby reducing communication burden while ensuring path-tracking accuracy.^10^ To mitigate model uncertainties and unpredictable disturbances, Jiang et al. developed a minimal learning parameter method for estimating and compensating for their effects on the USV system, ensuring the closed-loop system achieves uniform ultimate boundedness.^11^ Zhou et al. utilized fuzzy control techniques to approximate unknown external disturbances, and implemented an adaptive backstepping control for USV formation; however, backstepping methods often suffer from the “curse of dimensionality”.^12^ Additionally, Xiong et al. designed an extended state observer (ESO) that operates without any prior assumptions and incorporates an interactive identification algorithm to enhance the accuracy of disturbance estimation.^13^ For internal and external disturbances caused by multiple uncertainties, Huang et al. developed a reduced-order state observer and incorporated a nonlinear tracking differentiator to handle the “curse of dimensionality”.^14^ Despite these advances, existing disturbance-handling methods predominantly address matched disturbances, which share the same channel as the control input.^7^^,^^8^^,^^9^^,^^10^^,^^11^^,^^12^^,^^13^^,^^14^ However, these methods are fundamentally incapable of compensating for unmatched disturbances that act outside the control input channel. This gap highlights the need for innovative control strategies capable of mitigating the adverse effects of unmatched disturbances on USV trajectory tracking systems.

Unmatched disturbances in USVs mainly arise from sensor errors due to limited measurement accuracy, unmodeled dynamics that cause deviations between the actual system and the nominal model, and forces or moments induced by sudden environmental changes that are orthogonal to the main propulsion direction and thus cannot be compensated in real time by the primary thrust. These disturbances introduce significant noise into the system, potentially destabilizing it and rendering control algorithms designed solely for matched disturbances unsuitable for practical applications. Yao et al. addressed unmatched disturbances caused by position and velocity measurement deviations by designing a backstepping-based disturbance attenuation control strategy, enhancing system robustness.^15^ Wang et al. combined the Takagi-Sugeno fuzzy logic with an integral sliding-mode switching surface to ensure that the sliding motion of system states was influenced only by unamplified unmatched disturbances.^6^ However, it failed to effectively eliminate the influence of unmatched disturbances on the USV system. Given the limited studies on handling unmatched disturbances in USVs, potential solutions can be explored within the framework of nonlinear control theory. Yang et al. designed a disturbance observer that effectively eliminated the adverse effects of unmatched disturbances without relying on known disturbance bounds, significantly reducing system chattering.^16^ For *n*-order systems with matched and unmatched disturbances as well as model uncertainties, Hou et al. constructed a sliding mode control (SMC) approach integrating a nonlinear disturbance observer, which offered superior disturbance rejection compared to conventional SMC.^17^ Zhou et al. unified multiple unmatched disturbances into an equivalent total matched disturbance, constructing a linear ESO for non-integral chained nonlinear systems to improve both transient and steady-state performance.^18^ Moreover, Gandhi et al. combined the traditional ESO and the generalized ESO to design a hybrid ESO, enhancing the feasibility and applicability of control algorithms in non-integral chained nonlinear systems subject to multi-channel matched and unmatched disturbances.^19^ The suppression of unmatched disturbances in USVs is challenging due to their action outside control channels and the resulting degradation of closed-loop stability and control performance. The nonlinear control strategies present diverse possibilities for mitigating the adverse effects of unmatched disturbances in USV systems.^16^^,^^17^^,^^18^^,^^19^

Input saturation constitutes another significant challenge for the control performance of USV systems due to the physical constraints of thrusters, which cannot provide infinite force or moment. Nevertheless, the control strategies largely neglect this practical constraint.^3^^,^^5^^,^^6^^,^^8^^,^^9^^,^^10^^,^^11^^,^^14^ To tackle the intricacies introduced by complex uncertainties and input saturation in USV path tracking, Yu et al. devised an auxiliary system to compensate for saturation effects, presenting a finite-time control approach that guarantees the convergence of all system errors to a neighborhood of the origin within a finite time.^20^ Sun et al. introduced a guidance trajectory to prevent input saturation and used an approximate saturation function to mitigate oscillations arising from the sign function in conventional SMC.^21^ Hu et al. formulated an auxiliary dynamic filter to mitigate saturation effects, combining it with the dynamic event-triggering mechanism and fuzzy logic to propose an output feedback control scheme, thereby ensuring semi-global stability of the closed-loop system.^22^ However, strategies in Yu et al., Sun et al. and Hu et al. introduce the non-smooth behavior at saturation corners, which is detrimental to propulsion systems. To address this issue, Zhu et al. proposed a Gaussian error function-based smooth saturation function to approximate the non-smooth saturation nonlinearity, achieving smooth control inputs.^23^ Notably, existing methods for addressing input saturation inherently introduce discrepancies between the commanded and actual inputs, thereby requiring additional compensatory control strategies to account for these discrepancies, which increase the overall complexity of control design.^20^^,^^21^^,^^22^^,^^23^

It is important to emphasize that SMC has been widely applied to USV trajectory tracking due to its fast response, structural simplicity, and strong robustness. However, most existing SMC-based trajectory tracking methods rely on prior knowledge of the upper bounds of system disturbances or uncertainties.^24^^,^^25^ In practical engineering applications, such prior knowledge are unrealistic and may result in overestimated control gains, leading to unnecessary chattering. To address this issue, several nonlinear methods have been incorporated into SMC trajectory tracking methods for USVs.^26^^,^^27^^,^^28^ While these methods allow for dynamic adjustment of control gains, the convergence region and time of convergence for trajectory tracking errors often rely on the unknown upper thresholds of lumped disturbances. Thus, a critical challenge arises: how to design a trajectory tracking control strategy that does not rely on prior knowledge of lumped disturbance bounds, while simultaneously addressing both unmatched and matched disturbances, as well as input saturation constraints.

Inspired by the aforementioned control strategies, this work proposes two composite disturbance observer-based SMC strategies for the USV subject to unmatched disturbances, matched disturbances, and input saturation constraints. The main innovations are as follows.

(1) A composite disturbance observer is designed to effectively estimate both matched and unmatched disturbances, with the estimation error being bounded. Moreover, the observer features a simple structure and requires few tuning parameters.
(2) By introducing an auxiliary dynamic system, a composite disturbance observer-based SMC strategy is proposed. This strategy guarantees that the system states converge to a designated region while avoiding input saturation.
(3) An improved composite disturbance observer-based SMC strategy is proposed by incorporating a positive semi-definite barrier (PSDB) function. This strategy eliminates the need for auxiliary systems to compensate for input deviations, guarantees that finite-time convergence of the system is independent of unknown upper bounds of lumped disturbances, and prevents overestimation of control gains.

This paper is organized as follows: Section 2 formulates the problem, stating the requisite assumptions and lemmas. Section 3 details the main results, comprising the composite disturbance observer, the SMC strategy, and the improved SMC strategy. Section 4 offers numerical simulations across multiple scenarios to validate the proposed algorithms. Section 5 concludes the paper and sketches potential avenues for future research.

*Notations:* |·| is the absolute value function; ‖·‖ indicates the Euclidean norm; R represents the set of all real numbers; *λ*_*min*_(·) refers to the minimum eigenvalue; *sign*(·) stands for the sign function; For $x={\left[x_{1},x_{2},\cdots ,x_{n}\right]}^{T}$, $\text{sgn}\left(x\right)={\left[\text{sign}\left(x_{1}\right),\text{sign}\left(x_{2}\right),\cdots ,\text{sign}\left(x_{n}\right)\right]}^{T}$; *min*(·) signifies the minimum value in (·); *max*(·) denotes the maximum value in (·).

### Design

#### Problem formulation

The kinematic and dynamic mathematical models of a fully-actuated USV can be expressed as

(Equation 1) $$\dot{\eta }=J\left(\psi \right)\nu +d_{u}\left(t\right)$$

(Equation 2) $$M\dot{\nu }=-C\left(v\right)v-D\left(v\right)v+\tau +d_{m}\left(t\right)$$

where $\eta ={\left[x,y,\psi \right]}^{T}\in R^{3\times 1}$ stands for the position vector, defined by the position coordinates (*x*,*y*) and the yaw angle *ψ*∈[0,2*π*]; $v={\left[u,\upsilon ,r\right]}^{T}\in R^{3\times 1}$ represents the velocity vector, including the surge velocity *u*, sway velocity *υ*, and yaw rate *r*; *J*(*ψ*) = [*cos*(*ψ*),-*sin*(*ψ*),0;*sin*(*ψ*),*cos*(*ψ*),0;0,0,1] is the rotation matrix, which satisfies the relations *J*^*T*^(*ψ*) = *J*^−1^(*ψ*) and ‖*J*(*ψ*)‖ = 1; $M\in R^{3\times 3}$ is the positive definite and symmetric inertia matrix; $C\in R^{3\times 3}$ represents the Coriolis and centripetal force matrix; $D\in R^{3\times 3}$ is the linear damping matrix; $\tau ={\left[\tau_{1},\tau_{2},\tau_{3}\right]}^{T}\in R^{3\times 1}$ is the actual control input signal vector, including the equivalent force and moment provided by the propulsion system, consisting of the surge control force *τ*_1_, sway control force *τ*_2_, and yaw control moment *τ*_3_; $d_{u}\left(t\right)\in R^{3\times 1}$ represents the unmatched disturbances, which operate on channels distinct from the control input *τ*; $d_{m}\left(t\right)\in R^{3\times 1}$ represents the matched disturbances induced by environmental factors such as wind, waves, and currents.

Due to the physical characteristics of thrusters, the control inputs of the USV system are subject to symmetric saturation constraints, which can be described as

(Equation 3) $$\tau_{i}=\left\{ \begin{array}{l} \tau_{Mi}sign\left(\tau_{ci}\right),if\left|\tau_{ci}\right|\geq \left|\tau_{Mi}\right|\\ \tau_{ci},if\left|\tau_{ci}\right|\leq \left|\tau_{Mi}\right| \end{array}\right.,i=1,2,3$$

where $\tau_{c}={\left[\tau_{c1},\tau_{c2},\tau_{c3}\right]}^{T}$ is the commanded control signal, and *τ*_*Mi*_ represents the saturation amplitude of the system. The discrepancy between the commanded and actual control input signals can be represented as Δ*τ* = *τ*-*τ*_*c*_.

Assumption 1. The unmatched and matched disturbances are unknown but bounded, and their time derivatives are also bounded, as specified below:

(Equation 4) $$\left|d_{ui}\right|\leq \mu_{1i},\left|{\dot{d}}_{ui}\right|\leq \mu_{2i},\left|d_{mi}\right|\leq \mu_{3,i},\left|{\dot{d}}_{mi}\right|\leq \mu_{4,i},i=1,2,3$$

where *μ*_*j*,*i*_,*j* = 1,2,3,4 are unknown positive constants.

Lemma 1.^29^ For any (m,n)∈R and *σ* > 0, the following inequality holds:

(Equation 5) $$mn\leq \frac{\sigma^{k}}{k}{\left|m\right|}^{k}+\frac{\sigma^{-g}}{g}{\left|n\right|}^{g}$$

where *k* > 1, *g* > 1 and (*k*-1)(*g*-1) = 1.

Consider the nonlinear system described as

(Equation 6) $$\dot{x}=f\left(x\right),f\left(0\right)=0,x\left(0\right)=0$$

where $f:U_{0}\to R^{n}$ is a continuous function mapping in a neighborhood of the origin. When a Lyapunov function *V*(*x*) is selected such that *V*(*x*) = 0⇔*x* = 0, then the following lemmas hold.

Lemma 2.^30^ If the time derivative of the Lyapunov function *V*(*x*) satisfies the following inequality:

(Equation 7) $$\dot{V}\left(x\right)\leq -CV^{z}\left(x\right)$$

where *C* > 0 and 0 < *z* < 1, the system exhibits finite-time stability. The convergence time *T*_1_ is bounded by

(Equation 8) $$T_{1}\leq \frac{1}{C\left(1-z\right)}V^{1-z}\left(x_{0}\right)$$

where *V*(*x*_0_) represents the initial value of *V*(*x*).

Lemma 3.^31^ If the time derivative of the Lyapunov function *V*(*x*) satisfies the following inequality:

(Equation 9) $$\dot{V}\left(x\right)\leq -CV^{z}\left(x\right)+\delta$$

where *C* > 0, 0 < *z* < 1 and 0<*δ*<*∞*, the system (6) is practically finite-time stable. The convergence time *T*_2_ is bounded by

(Equation 10) $$T_{2}\leq \frac{1}{C\theta \left(1-z\right)}V^{1-z}\left(x_{0}\right)$$

where 0<*θ* < 1; *V*(*x*_0_) represents the initial value of *V*(*x*).

Lemma 4.^32^ If there exist constants *γ*_1_,*γ*_2_,⋯,*γ*_*l*_ > 0 and 0≤ƛ≤1, then the following inequality holds:

(Equation 11) $$\left(\gamma_{1}^{ƛ}+\gamma_{2}^{ƛ}+\cdots +\gamma_{l}^{ƛ}\right)\geq {\left(\gamma_{1}+\gamma_{2}+\cdots +\gamma_{l}\right)}^{ƛ}$$

Lemma 5.^33^ A PSDB function can be defined as

(Equation 12) $$L_{b}\left(\vartheta \right)=\frac{\left|\vartheta \right|}{\kappa -\left|\vartheta \right|}$$

where *κ* is a positive constant; *ϑ* is a variable with *ϑ*∈[-*κ*,*κ*]. *L*_*b*_(*ϑ*) has following properties: (1) *L*_*b*_(*ϑ*) is continuous and non-negative on the interval [-*κ*,*κ*], and it is monotonically increasing on [0,*κ*]; (2) $\lim_{\kappa \to \left|\vartheta \right|}L_{b}\left(\vartheta \right)\to \infty$; (3) *L*_*b*_(*ϑ*) has a unique minimum value *L*_*b*_(0) = 0 at *ϑ* = 0.

## Results

This section first formulates a composite disturbance observer to estimate both matched and unmatched disturbances. Subsequently, an SMC strategy is proposed to ensure the trajectory tracking error remains within a specified bound. Furthermore, an improved SMC strategy is developed that eliminates the need for unknown upper bounds of lumped disturbances and achieves smooth control inputs for the system.

### A composite disturbance observer

Define $x_{1}=e=\eta -\eta_{d}\in R^{3\times 1}$ and $x_{2}=J\left(\psi \right)v-{\dot{\eta }}_{d}\in R^{3\times 1}$, where *e* = *η*-*η*_*d*_ is the tracking error and *η*_*d*_ is the desired trajectory. Then, a new error dynamic system can be derived, which is expressed as

(Equation 13) $$\dot{x}=f\left(x\right)+g\left(x\right)+d$$

where $x={\left[x_{1}^{T},x_{2}^{T}\right]}^{T}$; $f\left(x\right)={\left[x_{2}^{T},0_{1\times 3}\right]}^{T}$; *g*(*x*) = [0_3×1_;*bτ*_*c*_]; *b* = *J*(*ψ*)*M*^−1^; $d={\left[d_{1}^{T},d_{2}^{T}\right]}^{T}$ is the composite disturbance vector; *d*_1_ = *d*_*u*_; *d*_2_ is the total matched disturbance and $d_{2}=J\left(\psi \right)M^{-1}\left(-C\left(\nu \right)-D\left(\nu \right)\right)\nu +\dot{J}\left(\psi \right)\nu +J\left(\psi \right)M^{-1}\left(d_{m}+\Delta \tau \right)-{\ddot{\eta }}_{d}$; $x_{j}={\left[x_{j1},x_{j2},x_{j3}\right]}^{T}$ and $d_{j}={\left[d_{j1},d_{j2},d_{j3}\right]}^{T},j=1,2$.

The composite disturbance observer is designed as

(Equation 14) $$\left\{ \begin{array}{l} \dot{\zeta }=-L\zeta -L\left(Lx+f\left(x\right)+g\left(x\right)\right)\\ \hat{d}=\zeta +Lx \end{array}\right.$$

where $\zeta \in R^{6\times 1}$ denotes the observer state; $L=\left[L_{1},0_{3\times 3};0_{3\times 3},L_{2}\right]\in R^{6\times 6}$; $L_{1}\in R^{3\times 3}$ and $L_{2}\in R^{3\times 3}$ are symmetric positive definite gain matrices; $\hat{d}={\left[{\hat{d}}_{1}^{T},{\hat{d}}_{2}^{T}\right]}^{T}$ is the estimate of *d* with ${\hat{d}}_{j}={\left[{\hat{d}}_{j1},{\hat{d}}_{j2},{\hat{d}}_{j3}\right]}^{T},j=1,2$.

Theorem 1. For the error dynamic system (13) subject to both unmatched and matched disturbances, the composite disturbance observer (14) ensures that the disturbance estimation errors are uniformly ultimately bounded, provided that appropriate observer gains *L*_1_ and *L*_2_ are chosen.

Proof 1. The estimation error is defined as $\tilde{d}=d-\hat{d}={\left[{\tilde{d}}_{1}^{T},{\tilde{d}}_{2}^{T}\right]}^{T}$ with ${\tilde{d}}_{j}={\left[{\tilde{d}}_{j1},{\tilde{d}}_{j2},{\tilde{d}}_{j3}\right]}^{T},j=1,2$, and according to (Equations 13) and (14), we can obtain

(Equation 15) $$\dot{\hat{d}}=L\left(d-\hat{d}\right)$$

(Equation 16) $$\dot{\tilde{d}}=\Phi \tilde{d}+\dot{d}$$

where Φ = [-*L*_1_,0_3×3_;0_3×3_,-*L*_2_]; $\dot{d}={\left[{\dot{d}}_{1}^{T},{\dot{d}}_{2}^{T}\right]}^{T}$ and ${\dot{d}}_{j}={\left[{\dot{d}}_{j1},{\dot{d}}_{j2},{\dot{d}}_{j3}\right]}^{T},j=1,2$. By assumption 1 and (Equation 13), the Euclidean norm of $\dot{d}$ satisfies

(Equation 17) $$\left\|\dot{d}\right\|\leq d_{0}$$

where *d*_0_ is an unknown constant.

For the error dynamic system (13), suitable observer gains *L*_1_ and *L*_2_ can always be selected such that the eigenvalues of Φ are located in the left-half plane. Consequently, there exists a positive definite matrix $P\in R^{6\times 6}$ satisfying

(Equation 18) $$\Phi^{T}P+P\Phi =-Q$$

where $Q\in R^{6\times 6}$ is an arbitrary positive definite matrix.

A Lyapunov candidate function $V\left(\tilde{d}\right)$ is constructed as

(Equation 19) $$V\left(\tilde{d}\right)={\tilde{d}}^{T}P\tilde{d}$$

By combining (Equations 17) and (18) and taking the time derivative of Equation 19, we have

(Equation 20) $$\dot{V}\left(\tilde{d}\right)={\tilde{d}}^{T}\left(\Phi^{T}P+P\Phi \right)\tilde{d}+2{\tilde{d}}^{T}P\dot{d}\leq -{\tilde{d}}^{T}Q\tilde{d}+2\left\|\tilde{d}\right\|\left\|P\right\|\left\|\dot{d}\right\|\leq -\left\|\tilde{d}\right\|\left(\lambda_{\min}\left(Q\right)\left\|\tilde{d}\right\|-2d_{0}\left\|P\right\|\right)$$

Thus, after sufficient time, the estimation error $\tilde{d}$ will become uniformly ultimately bounded and can be expressed as

(Equation 21) ‖d˜‖≤2d0‖P‖λmin(Q)

Furthermore, based on $\tilde{d}={\left[{\tilde{d}}_{1}^{T},{\tilde{d}}_{2}^{T}\right]}^{T}$, it can be reasonably inferred that the estimation errors of *d*_1_ and *d*_2_ are ultimately bounded, as described by

(Equation 22) $$\left\|{\tilde{d}}_{1}\right\|\leq \varepsilon_{1},\left\|{\tilde{d}}_{2}\right\|\leq \varepsilon_{2}$$

where *ε*_1_ and *ε*_2_ are unknown positive constants. This concludes the proof of Theorem 1.

Remark 1. Unlike the disturbance handling methods in Liu et al.,^8^ Deng et al.,^9^ Jiang et al.,^11^ Xiong et al.,^13^ and Huang et al.,^14^ the designed nonlinear disturbance observer (14) estimates and compensates for both unmatched and matched disturbances, thereby significantly enhancing the system’s control performance while requiring fewer tuning parameters. Notably, the rate of convergence for the estimation error is directly influenced by the observer gains *L*_1_ and *L*_2_. While larger gains ensure rapid convergence to a smaller bounded region, they may also amplify system noise. Thus, a balance between control accuracy and observation quality must be considered when selecting the observer gains.

### The sliding mode trajectory tracking control strategy

**Figure 1:**
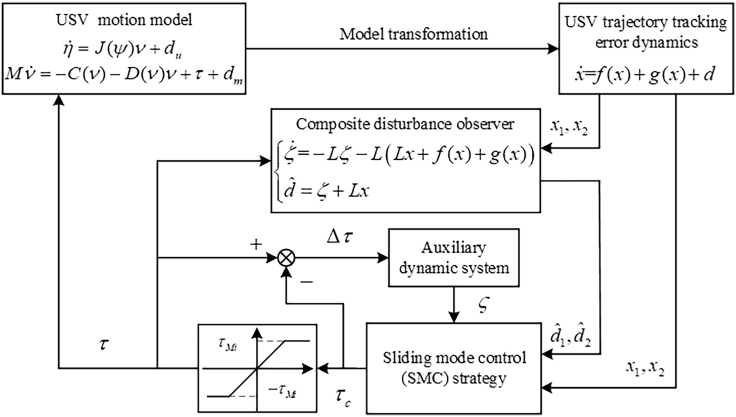
The control framework of the SMC system

This section presents an SMC strategy for USV trajectory tracking, integrating the previously designed composite disturbance observer with an auxiliary dynamic system. The control framework of the SMC system is depicted in Figure 1.

### An auxiliary dynamic system

When the output of the trajectory tracking control law is processed through the saturation model (3), a discrepancy arises between the commanded and actual control signals. However, the saturation model (3) cannot compensate for this discrepancy, potentially leading to system chattering or instability. To resolve this, an auxiliary dynamic system $\varsigma ={\left[\varsigma_{1},\varsigma_{2},\varsigma_{3}\right]}^{T}\in R^{3\times 1}$ is constructed, which is constructed as follows:

(Equation 23) $$\dot{\varsigma }=-M^{-1}K_{0}\varsigma +M^{-1}\Delta \tau$$

where $K_{0}\in R^{3\times 3}$ is a symmetric positive definite matrix.

A Lyapunov candidate function *V*_*ς*_ is chosen as

(Equation 24) $$V_{\varsigma }=\frac{1}{2}\varsigma^{T}M\varsigma$$

Differentiating (Equation 24) with respect to time, according to Lemma 1, yields:

(Equation 25) $${\dot{V}}_{\varsigma }=\varsigma^{T}M\dot{\varsigma }\leq -\lambda_{\min}\left(\left(K_{0}-\frac{I_{3\times 3}}{2}\right)M^{-1}\right)\varsigma^{T}M\varsigma +\frac{1}{2}{\left\|\Delta \tau \right\|}^{2}\leq -2a_{1}V_{\varsigma }+{Ν}_{1}$$

where $a_{1}=\lambda_{\min}\left(\left(K_{0}-\frac{I_{3\times 3}}{2}\right)M^{-1}\right)$; ${Ν}_{1}=\frac{1}{2}{\left\|\Delta \tau \right\|}^{2}$. Then, the stability of the auxiliary system is ensured if the design parameter satisfies the following condition:

(Equation 26) $$\lambda_{\min}\left(K_{0}\right)-\frac{1}{2}>0$$

Then, solving (Equation 25) yields

(Equation 27) $$\left\|\varsigma \right\|\leq \sqrt{\frac{{Ν}_{1}}{2a_{1}}+\left[V_{\varsigma }\left(0\right)-\frac{{Ν}_{1}}{2a_{1}}\right]e^{-2a_{1}t}}$$

where *V*_*ς*_(0) represents the initial value of *V*_*ς*_.

From Equation 27, it follows that ‖*ς*‖ remains ultimately uniformly bounded. Furthermore, it can be shown that |*ς*_*i*_| is bounded satisfies:

(Equation 28) $$\left|\varsigma_{i}\right|\leq \sigma_{i},i=1,2,3$$

where *σ*_*i*_ is an unknown positive constant.

### An SMC strategy

In this subsection, an SMC strategy with the composite disturbance observer (14) and the auxiliary dynamic system (23) is proposed for the USV subject to unmatched disturbances, matched disturbances, and input saturation.

The sliding mode surface $s_{1}\in R^{3\times 1}$ and an auxiliary state ${\bar{s}}_{1}\in R^{3\times 1}$ are defined as

(Equation 29) $$\left\{ \begin{array}{l} s_{1}={\bar{s}}_{1}-\Pi {\bar{s}}_{1}\left(0\right)\\ {\bar{s}}_{1}=x_{2}+\rho x_{1}+{\hat{d}}_{1} \end{array}\right.$$

where Π = *diag*([Π_1_,Π_2_,Π_3_]) with $\Pi_{i}=e^{-\alpha_{i}t}$ and *α*_*i*_ > 0,*i* = 1,2,3; *ρ* = *diag*([*ρ*_1_,*ρ*_2_,*ρ*_3_]) is a positive definite matrix and *ρ*_*i*_≠*α*_*i*_; ${\bar{s}}_{1}\left(0\right)$ is the initial value of the auxiliary state ${\bar{s}}_{1}$; $s_{1}={\left[s_{11},s_{12},s_{13}\right]}^{T}\in R^{3\times 1}$; ${\bar{s}}_{1}={\left[{\bar{s}}_{11},{\bar{s}}_{12},{\bar{s}}_{13}\right]}^{T}\in R^{3\times 1}$.

The SMC strategy is proposed as

(Equation 30) $$\tau_{c}=-b^{-1}\left(\rho \left(x_{2}+{\hat{d}}_{1}\right)+{\hat{d}}_{2}+\alpha \Pi {\bar{s}}_{1}\left(0\right)\right)-b^{-1}\left(K_{1}s_{1}+K_{2}\text{sgn}\left(s_{1}\right)\right)+K_{0}\varsigma$$

where *α* = *diag*([*α*_1_,*α*_2_,*α*_3_]), *K*_1_ = *diag*([*K*_11_,*K*_12_,*K*_13_]), and *K*_2_ = *diag*([*K*_21_,*K*_22_,*K*_23_]) are positive definite matrices.

Theorem 2. Under Assumption 1, consider the error dynamic system (13). If the SMC trajectory tracking strategy (30), incorporating the composite disturbance observer (14) and the auxiliary dynamic system (23), is implemented with appropriately selected design parameters, the sliding mode variable *s*_1*i*_ will converge to zero in finite time. Furthermore, system states *x*_1*i*_ and *x*_2*i*_ will converge to the designated regions |*x*_1*i*_|≤*ε*_1_/*ρ*_*i*_ and |*x*_2*i*_|≤2*ε*_1_+*μ*_1*i*_,*i* = 1,2,3, respectively.

Proof 2. In the light of Equations 13, 15, and 30 and $\dot{\Pi }=-\alpha \Pi$, the time derivative of Equation 29 yields:

(Equation 31) $${\dot{s}}_{1}={\dot{\bar{s}}}_{1}+\alpha \Pi {\bar{s}}_{1}\left(0\right)=-K_{1}s_{1}-K_{2}\text{sgn}\left(s_{1}\right)+h\left(t\right)$$

where $h\left(t\right)=\left(\rho +L_{1}\right){\tilde{d}}_{1}+{\tilde{d}}_{2}+J\left(\psi \right)M^{-1}K_{0}\varsigma$ is the lumped disturbance, and $h\left(t\right)={\left[h_{1}\left(t\right),h_{2}\left(t\right),h_{3}\left(t\right)\right]}^{T}\in R^{3\times 1}$.

By defining ϒ = *J*(*ψ*)*M*^−1^*K*_0_*ς* and referring to (Equation 28), it can be reasonably deduced that $\left|{ϒ}_{i}\right|\leq {\bar{\delta }}_{i}$, where ${\bar{\delta }}_{i},i=1,2,3$ are unknown constants. Thus, from Equation 22 and $\left|{ϒ}_{i}\right|\leq {\bar{\delta }}_{i}$, it follows that |*h*_*i*_(*t*)| is also ultimately bounded and |*h*_*i*_(*t*)|≤*h*_*Mi*_, where $h_{Mi}=\left\|\rho +L_{1}\right\|\varepsilon_{1}+\varepsilon_{2}+{\bar{\delta }}_{i},i=1,2,3$.

Chosen a Lyapunov candidate function as

(Equation 32) $$V_{1i}\left(s_{1i}\right)=\frac{1}{2}s_{1i}^{2},i=1,2,3$$

Taking the time derivative of Equation 32, we have

(Equation 33) $${\dot{V}}_{1i}\left(s_{i1}\right)=s_{1i}{\dot{s}}_{1i}=s_{1i}\left(-K_{1i}s_{1i}-K_{2i}sign\left(s_{1i}\right)+h_{i}\left(t\right)\right)\leq h_{Mi}\left|s_{1i}\right|-K_{1i}s_{1i}^{2}-K_{2i}\left|s_{1i}\right|\leq -\left(-h_{Mi}+K_{2i}\right)\left|s_{1i}\right|$$

Therefore, guaranteeing the finite-time stability of the closed-loop system requires the following condition:

(Equation 34) $$K_{2i}\geq h_{Mi}$$

Thus, (Equation 33) can be rewritten as

(Equation 35) $${\dot{V}}_{1i}\left(s_{i1}\right)\leq -c_{1i}V_{1i}^{\frac{1}{2}}$$

where $c_{1i}=\sqrt{2}\left(-h_{Mi}+K_{2i}\right),i=1,2,3$.

By applying Lemma 2, the sliding mode variable *s*_1*i*_ converges to zero in finite time, with the convergence time *T*_1*i*_ satisfying

(Equation 36) $$T_{1i}\leq \frac{2}{c_{1i}}V_{1i}^{\frac{1}{2}}\left(s_{1i}\left(0\right)\right)$$

where *V*_1*i*_(*s*_1*i*_(0)) denotes the initial value of *V*_1*i*_(*s*_*i*1_).

From Equation 29, one obtains

(Equation 37) $$s_{1i}={\dot{x}}_{1i}+\rho_{i}x_{1i}-{\tilde{d}}_{1i}-e^{-\alpha_{i}t}{\bar{s}}_{i}\left(0\right)$$

Consider the condition of *s*_1*i*_ = 0, based on Equations 13 and 29, we have

(Equation 38) $${\dot{x}}_{1i}=-\rho_{i}x_{1i}+{\tilde{d}}_{1i}+e^{-\alpha_{i}t}{\bar{s}}_{i}\left(0\right)$$

Then, integrating (Equation 38) with respect to time over the interval [0,+∞) yields

(Equation 39) $$x_{1i}=\int_{0}^{t}\left({\bar{s}}_{1i}\left(0\right)e^{-\alpha_{i}\chi }-{\tilde{d}}_{1i}\left(\chi \right)\right)e^{-\rho_{i}\left(t-\chi \right)}d\chi +x_{1i}\left(0\right)e^{-\rho_{i}t}$$

Combining (Equation 22), it can be concluded that $\left|{\tilde{d}}_{1i}\right|\leq \varepsilon_{1}$, and it follows that

(Equation 40) $$\left|x_{1i}\right|\leq \left|x_{1i}\left(0\right)\right|e^{-\rho_{i}t}+\left|{\bar{s}}_{i}\left(0\right)e^{-\rho_{i}\chi }\int_{0}^{t}e^{-\left(\alpha_{i}-\rho_{i}\right)\chi }d\chi \right|+\left|\varepsilon_{1}\int_{0}^{t}e^{-\rho_{i}\left(t-\chi \right)}d\chi \right|=\left|x_{1i}\left(0\right)\right|e^{-\rho_{i}t}+\frac{{\bar{s}}_{1i}\left(0\right)e^{-\rho_{i}\chi }}{\alpha_{i}-\rho_{i}}\left(1-e^{-\left(\alpha_{i}-\rho_{i}\right)t}\right)+\frac{\varepsilon_{1}}{\rho_{i}}\left(1-e^{-\rho_{i}t}\right)$$

Since *ρ*_*i*_ > 0, and according to (Equation 40), we can derive that

(Equation 41) $$\left|x_{1i}\right|\leq \frac{\varepsilon_{1}}{\rho_{i}},t\to \infty$$

When *t*→*∞*, in view of Equation 40 and ${\tilde{d}}_{1i}=d_{1i}-{\hat{d}}_{1i}$, it follows that

(Equation 42) $$\left|x_{2i}\right|\leq \rho_{i}\left|x_{1i}\right|+\left|d_{1i}-{\tilde{d}}_{1i}\right|+\Pi_{i}\left|{\bar{s}}_{1i}\left(0\right)\right|\leq 2\varepsilon_{1}+\mu_{1i}$$

Thus, the system states *x*_1_ and *x*_2_ are ensured to converge to a neighborhood of the origin. Combining (Equation 41) and *x*_1_ = *e*, it can be deduced that the tracking error is ultimate uniform bounded. Moreover, by appropriately selecting the control gains *ρ*_1_,*α*_1_,*L*_1_,*L*_2_,*K*_0_,*K*_1_ and *K*_2_, the trajectory tracking error can be made to converge to a neighborhood of the origin as small as possible. This concludes the proof of Theorem 2.

Remark 3. It is crucial to acknowledge that the proposed SMC strategy (30) exhibits two issues that need to be addressed:

#### Issue 1

The introduction of the auxiliary dynamic system (23) increases the complexity of the stability proof and imposes an additional constraint on the selection of the control gain K_2_, resulting in a more conservative control design. Furthermore, as can be observed from the saturation model (3), the control input becomes non-smooth at the saturation corners, which hinders the realization of smooth control inputs, posing challenges for the propulsion system of the USV.

#### Issue 2

As indicated in Equation 34, the selection of control gain K_2_ requires prior knowledge of the upper bounds of the lumped disturbance h(t) and relevant information about the auxiliary dynamic system. Generally, such information is difficult to obtain directly. Consequently, to counteract the adverse effects of both matched and unmatched disturbances as well as input discrepancies, the control gains may be overestimated, potentially leading to unnecessary chattering in the system.

To address the above issues, in the next subsection, we will design an improved sliding mode trajectory tracking control strategy, which does not require prior knowledge of the upper bounds of the lumped disturbances and does not involve the introduction of an additional auxiliary dynamic system.

### An improved SMC strategy

**Figure 2:**
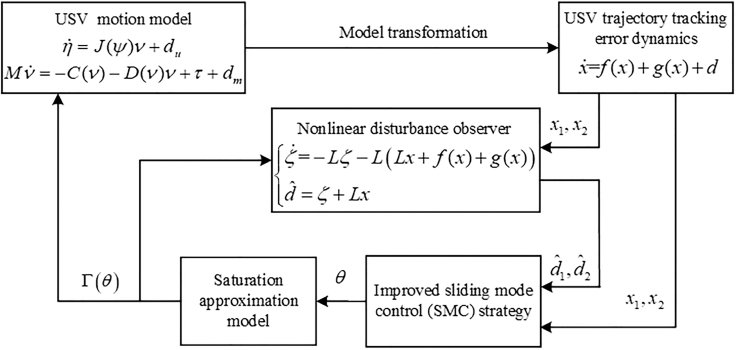
The control framework of the improved SMC system

In this subsection, we first construct a saturation approximation model to handle input saturation. Then, by introducing a PSDB function to prevent overestimation of control gains, an improved SMC strategy is proposed for the USV subject to unmatched disturbances, matched disturbances, and input saturation. The control framework of the improved SMC system is depicted in Figure 2.

To ensure smooth characteristics of control inputs, a hyperbolic tangent function is applied to construct a smooth saturation approximation model with an auxiliary control system, as expressed by^34^

(Equation 43) $$\tau_{i}=\Gamma_{i}\left(\theta_{i}\right)=\tau_{Mi}\;\tanh\left(\frac{\theta_{i}}{\tau_{Mi}}\right),i=1,2,3$$

where $\Gamma \left(\theta \right)={\left[\Gamma_{1}\left(\theta_{1}\right),\Gamma_{2}\left(\theta_{2}\right),\Gamma_{3}\left(\theta_{3}\right)\right]}^{T}$ denotes the saturation approximation model; $\theta ={\left[\theta_{1},\theta_{2},\theta_{3}\right]}^{T}$ is the auxiliary control system. Moreover, Γ(θ) has the properties $\left|\Gamma_{i}\left(\theta_{t}\right)\right|=\tau_{Mi}\left|\tanh\left(\frac{\theta_{i}}{\tau_{Mi}}\right)\right|\leq \tau_{Mi}$ and $0<\frac{\partial \Gamma_{i}\left(\theta_{i}\right)}{\partial \theta_{i}}=\frac{4}{{\left(e^{\left(\theta_{i}/\tau_{Mi}\right)}+e^{\left(-\theta_{i}/\tau_{Mi}\right)}\right)}^{2}}\leq 1$.

The auxiliary control system is designed as

(Equation 44) $$\dot{\theta }={Ν}^{-1}X$$

where $Ν=diag\left(\left[\frac{\partial \Gamma_{1}\left(\theta_{1}\right)}{\partial \theta_{1}},\frac{\partial \Gamma_{2}\left(\theta_{2}\right)}{\partial \theta_{2}},\frac{\partial \Gamma_{3}\left(\theta_{3}\right)}{\partial \theta_{3}}\right]\right)$; $Χ={\left[{Χ}_{1},{Χ}_{2},{Χ}_{3}\right]}^{T}\in R^{3\times 1}$ is the auxiliary control signal. Hence, the control design problem for strategy τ transforms into the design of the auxiliary signal Χ.

**Figure 3:**
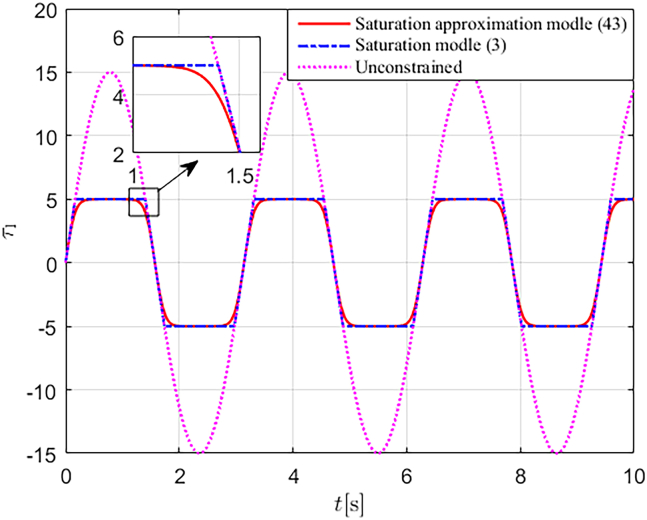
Saturation constraint curves of *τ*_1_

Let us consider the function τ_1_ = Γ_1_(θ_1_) as an example. When θ_1_(0) = 0, we design ${\dot{\theta }}_{1}=15\;\cos\;t$. The saturation amplitude of Γ1(θ1) is set as τ_M1_ = 5, and the saturation constraints of the function are handled using (Equation 3), (Equation 43), respectively. The simulation results, shown in Figure 3, indicate that the saturation approximation model (43) effectively approximates the saturation model (3) while ensuring smooth behavior at saturation corners.

In contrast to the saturation model (3), the saturation approximation model (43) aligns the commanded and actual control signals, thereby eliminating the need for additional auxiliary dynamic systems and simplifying the design of the control strategy. The modified error dynamic system is given by

(Equation 45) $$\dot{x}=f\left(x\right)+g\left(x\right)+d$$

where g(x) = [0_3×1_;bΓ(θ)] and $d_{2}=J\left(\psi \right)M^{-1}\left(-C\left(v\right)-D\left(v\right)\right)v+\dot{J}\left(\psi \right)v+J\left(\psi \right)M^{-1}d_{m}-{\ddot{\eta }}_{d}$, and the remaining symbols are consistent with those in the subsection 3.2.2.

The first sliding mode surface is defined identically to the one given in Equation 29. Additionally, we introduce a first-order low-pass filter as

(Equation 46) $$T_{1}{\dot{x}}_{d1}+x_{d1}=z_{1},x_{d1}\left(0\right)=z_{1}\left(0\right)$$

where $z_{1}\in R^{3\times 1}$ is the intermediate control vector; T_1_ > 0 is the time constant; $x_{d1}\in R^{3\times 1}$ is the filter’s state vector.

The filtering error vector $Y_{1}\in R^{3\times 1}$ is defined as

(Equation 47) $$Y_{1}=x_{d1}-z_{1}$$

Remark 3. The introduction of the state vector *x*_*d*1_ serves to replace the intermediate control vector *z*_1_, and ${\dot{x}}_{d1}=\left(z_{1}-x_{d1}\right)/T_{1}$ can be derived from.46 This approach effectively prevents the “curse of dimensionality” issue that would otherwise arise from differentiating the intermediate control vector during the subsequent stages of the control strategy design.

The second sliding mode surface $s_{2}={\left[s_{21},s_{22},s_{23}\right]}^{T}\in R^{3\times 1}$ is designed as

(Equation 48) $$s_{2}=\Gamma \left(\theta \right)-x_{d1}$$

From Equations 47 and 48, we obtain

(Equation 49) $$\Gamma \left(\theta \right)=s_{2}+Y_{1}+z_{1}$$

Using Equation 49, the time derivative of the first sliding mode surface *s*_1_ can be represented as

(Equation 50) $${\dot{s}}_{1}=b\Gamma \left(\theta \right)+d_{2}+\rho \left(x_{2}+d_{1}\right)+{\dot{\hat{d}}}_{1}+\alpha \Pi {\bar{s}}_{1}\left(0\right)=b\left(s_{2}+Y_{1}+z_{1}\right)+d_{2}+\rho \left(x_{2}+d_{1}\right)+{\dot{\hat{d}}}_{1}+\alpha \Pi {\bar{s}}_{1}\left(0\right)$$

By combining the PSDB function and the composite disturbance observer, the intermediate control vector *z*_1_ is designed as

(Equation 51) $$z_{1}=-b^{-1}\left(\rho \left(x_{2}+{\hat{d}}_{1}\right)+{\hat{d}}_{2}+\alpha \Pi {\bar{s}}_{1}\left(0\right)\right)-b^{-1}\left(K_{1}s_{1}+L_{b}\left(s_{1}\right)\text{sgn}\left(s_{1}\right)\right)$$

where *L*_*b*_(*s*_1_) = *diag*([*L*_*b*1_(*s*_11_),*L*_*b*2_(*s*_12_),*L*_*b*3_(*s*_13_)]) is the PSDB function and it can be described as

(Equation 52) $$L_{bi}\left(s_{1i}\right)=\frac{\left|s_{1i}\right|}{\kappa_{i}-\left|s_{1i}\right|},s_{1i}\in \left[-\kappa_{i},\kappa_{i}\right],i=1,2,3$$

where *κ*_*i*_ is a known positive real number. According to Lemma 5, the PSDB function prevents overestimation of the control gains and guarantees that the system states converge to a restricted region.

Substituting (Equation 51) into (Equation 50), we get

(Equation 53) $${\dot{s}}_{1}=b\left(s_{2}+Y_{1}+z_{1}\right)+d_{2}+\rho \left(x_{2}+d_{1}\right)+{\dot{\hat{d}}}_{1}+\alpha \Pi {\bar{s}}_{1}\left(0\right)=\rho {\tilde{d}}_{1}+{\tilde{d}}_{2}+L_{1}{\tilde{d}}_{1}-K_{1}s_{1}-L_{b}\left(s_{1}\right)\text{sgn}\left(s_{1}\right)+bs_{2}+bY_{1}=\bar{h}\left(t\right)-K_{1}s_{1}-L_{b}\left(s_{1}\right)\text{sgn}\left(s_{1}\right)+{Η}_{1}+{Η}_{2}$$

where $\bar{h}\left(t\right)=\left(\rho +L_{1}\right){\tilde{d}}_{1}+{\tilde{d}}_{2}$ is the lumped disturbance; ${Η}_{1}=bs_{2}={\left[{Η}_{11},{Η}_{12},{Η}_{13}\right]}^{T}$; ${Η}_{2}=bY_{1}={\left[{Η}_{21},{Η}_{22},{Η}_{23}\right]}^{T}$.

By applying (Equation 22), we deduce that $\left\|\bar{h}\left(t\right)\right\|\leq \left\|\rho +L_{1}\right\|\varepsilon_{1}+\varepsilon_{2}$ and $\left|{\bar{h}}_{i}\left(t\right)\right|\leq {\bar{h}}_{Mi}$, where ${\bar{h}}_{Mi},i=1,2,3$ are unknown constants.

To facilitate stability analysis, an auxiliary variable *ϕ*_*i*_ is introduced as

(Equation 54) $$\phi_{i}=\kappa_{i}\frac{\left|{\bar{h}}_{Mi}\right|}{1+\left|{\bar{h}}_{Mi}\right|}<\kappa_{i},i=1,2,3$$

A Lyapunov candidate function is constructed as

(Equation 55) $$V_{1i}\left(s_{1i}\right)=\frac{1}{2}s_{1i}^{2},i=1,2,3$$

According to (Equation 53) and Lemma 1, taking the time derivative of Equation 55 yields

(Equation 56) $${\dot{V}}_{1i}\left(s_{1i}\right)=s_{1i}\left({\bar{h}}_{i}\left(t\right)-K_{1i}s_{1i}-L_{bi}\left(s_{1i}\right)\text{sgn}\left(s_{1i}\right)+{Η}_{1i}+{Η}_{2i}\right)\leq -\left(K_{1i}-\frac{1}{2}\right)s_{1i}^{2}+{\bar{h}}_{Mi}\left|s_{1i}\right|-L_{bi}\left(s_{1i}\right)\left|s_{1i}\right|+s_{1i}{Η}_{1i}+\frac{1}{2}{Η}_{2i}^{2}$$

Thus, (Equation 56) can be rewritten as

(Equation 57) $${\dot{V}}_{1i}\left(s_{1i}\right)\leq -\left(K_{1i}-\frac{1}{2}\right)s_{1i}^{2}-\left(-{\bar{h}}_{Mi}+L_{bi}\left(s_{1i}\right)\right)\left|s_{1i}\right|+s_{1i}{Η}_{1i}+\frac{1}{2}{Η}_{2i}^{2}$$

According to (Equation 44), the derivative of the second sliding mode surface with respect to time is given by

(Equation 58) $${\dot{s}}_{2}=Ν\dot{\theta }-{\dot{x}}_{d1}=Χ-{\dot{x}}_{d1}$$

Thus, the auxiliary control signal is designed as

(Equation 59) $$Χ=-K_{2}s_{2}-b^{T}s_{1}-K_{3}\text{sgn}\left(s_{2}\right)+{\dot{x}}_{d1}$$

where *K*_3_ = *diag*([*K*_31_,*K*_32_,*K*_33_]) is a symmetric positive definite matrix.

In view of Equations 58 and 59, we deduce that

(Equation 60) $${\dot{s}}_{2}=-K_{2}s_{2}-b^{T}s_{1}-K_{3}\text{sgn}\left(s_{2}\right)$$

Choose a Lyapunov candidate function as

(Equation 61) $$V_{2i}\left(s_{2i}\right)=\frac{1}{2}s_{2i}^{2},i=1,2,3$$

Based on Equation 60, taking the time derivative of Equation 61 yields

(Equation 62) $${\dot{V}}_{2i}\left(s_{2i}\right)=s_{2i}\left(-K_{2i}s_{2i}-{Η}_{3i}-K_{3i}sign\left(s_{2i}\right)\right)\leq -K_{2i}s_{2i}^{2}-s_{2i}{Η}_{3i}-K_{3i}\left|s_{2i}\right|$$

where ${Η}_{3}=b^{T}s_{1}=\left[{Η}_{31},{Η}_{32},{Η}_{33}\right]\in R^{3\times 1}$.

Therefore, the design of the improved SMC strategy is now complete, and the following theorem can be derived.

Theorem 3. Under Assumption 1, consider the error dynamic system (13). If the improved sliding mode trajectory tracking control strategy for the USV is implemented using the composite disturbance observer (13), sliding surfaces (29) and (48), intermediate control vector (51), and auxiliary control signal (59) as defined in Equation 43, and if the design parameters are suitably selected, then the sliding mode variable *s*_1*i*_ will converge to the interval [-*κ*_*i*_,*κ*_*i*_] in finite time, while the system states *x*_1*i*_ and *x*_2*i*_ will converge to the designated regions |*x*_1*i*_|≤(*κ*_*i*_+*ε*_1_)/*ρ*_*i*_ and |*x*_2*i*_|≤2*κ*_*i*_+2*ε*_1_+*μ*_1*i*_,*i* = 1,2,3, respectively.

Proof 3. The Lyapunov candidate function for the entire closed-loop system is constructed as

(Equation 63) $$V\left(s_{1},s_{2}\right)=\sum_{i=1}^{3}V_{i}\left(s_{1i},s_{2i}\right)=\sum_{i=1}^{3}\left(V_{1i}\left(s_{1i}\right)+V_{2i}\left(s_{2i}\right)\right)$$

From Equations 57 and 62, we obtain

(Equation 64) $${\dot{V}}_{i}\left(s_{1i},s_{2i}\right)\leq -\left(K_{1i}-\frac{1}{2}\right)s_{1i}^{2}-\left(-{\bar{h}}_{Mi}+L_{bi}\left(s_{1i}\right)\right)\left|s_{1i}\right|+s_{1i}{Η}_{1i}+\frac{1}{2}{Η}_{2i}^{2}-K_{2i}s_{2i}^{2}-s_{2i}{Η}_{3i}-K_{3i}\left|s_{2i}\right|$$

Next, a Lyapunov candidate function for the filtering error is introduced as

(Equation 65) $$V_{y}=\frac{1}{2}Y_{1}^{T}Y_{1}$$

According to Equation 51, *z*_1_ is a function of the variable set $\left(s_{1},{\bar{s}}_{1},x_{2},{\hat{d}}_{1},{\hat{d}}_{2},L_{b}\right)$, and it can be obtained that

(Equation 66) $${\dot{Y}}_{1}={\dot{x}}_{d1}-{\dot{z}}_{1}=-\frac{Y_{1}}{T_{1}}+B\left(s_{1},{\bar{s}}_{1},x_{2},{\hat{d}}_{1},{\hat{d}}_{2},L_{b},{\dot{s}}_{1},{\dot{\bar{s}}}_{1},{\dot{x}}_{2},{\dot{\hat{d}}}_{1},{\dot{\hat{d}}}_{2},{\dot{L}}_{b}\right)$$

where $B\left(s_{1},{\bar{s}}_{1},x_{2},{\hat{d}}_{1},{\hat{d}}_{2},L_{b},{\dot{s}}_{1},{\dot{\bar{s}}}_{1},{\dot{x}}_{2},{\dot{\hat{d}}}_{1},{\dot{\hat{d}}}_{2},{\dot{L}}_{b}\right)=-{\dot{z}}_{1}$.

Consider that there exist bounded compact sets $\Omega_{1}=\left\{\left(s_{1},{\bar{s}}_{1},x_{2},{\hat{d}}_{1},{\hat{d}}_{2},L_{b},{\dot{s}}_{1},{\dot{\bar{s}}}_{1},{\dot{x}}_{2},{\dot{\hat{d}}}_{1},{\dot{\hat{d}}}_{2},{\dot{L}}_{b}\right)|\left\|{\dot{z}}_{1}\right\|\leq \omega_{0}\right\}$ and Ω_2_ = {(*s*_2_,*x*_1_,*Y*_1_)|*V*≤*ω*_1_} in the system, where *ω*_0_ and *ω*_1_ are both positive constants. Therefore, Ω_1_×Ω_2_ is also a bounded compact set. It can be reasonably deduced that there exists a bounded continuous function *B*_1_(·) such that $\left\|{\dot{Y}}_{1}+Y_{1}/T_{1}\right\|\leq B_{1}\left(s_{1},{\bar{s}}_{1},x_{1},x_{2},Y_{1},{\hat{d}}_{1},{\hat{d}}_{2},L_{b},{\dot{s}}_{1},{\dot{\bar{s}}}_{1},{\dot{x}}_{2},{\dot{\hat{d}}}_{1},{\dot{\hat{d}}}_{2},{\dot{L}}_{b}\right)$, and *B*_1_(·) has a maximum value Ζ > 0 in Ω_1_×Ω_2_, i.e., ‖*B*_1_(·)‖≤Ζ. Therefore, in the light of Equation 66 and Lemma 1, we have

(Equation 67) $${\dot{V}}_{y}=-\frac{Y_{1}^{T}Y_{1}}{T_{1}}+Y_{1}^{T}\left(\frac{Y_{1}}{T_{1}}+{\dot{Y}}_{1}\right)\leq -\left(\frac{1}{T_{1}}-\frac{1}{2}\right)Y_{1}^{T}Y_{1}+\frac{{Ζ}^{2}}{2}=-\beta V_{y}+\frac{{Ζ}^{2}}{2}$$

where $\beta =2\left(\frac{1}{T_{1}}-\frac{1}{2}\right)$. To ensure the boundedness of *V*_*y*_, the following condition must be satisfied:

(Equation 68) $$0<T_{1}<2$$

From Equations 65, 67, and 68, it follows that *Y*_1_ is uniformly ultimately bounded. Moreover, considering *b* = *J*(*ψ*)*M*^−1^ and ‖*J*(*ψ*)‖ = 1, it can be inferred that Η_2*i*_ is also bounded. Therefore, it can be expressed as

(Equation 69) $$\left|Y_{1i}\right|<\varphi_{i},\left|{Η}_{2i}\right|\leq {\bar{\varphi }}_{i}$$

where *φ*_*i*_ and ${\bar{\varphi }}_{i}$ are unknown positive constants.

Furthermore, to ensure finite-time convergence of the closed-loop system, the following condition must hold:

(Equation 70) $$K_{1i}-\frac{1}{2}>0$$

Since *s*_2*i*_Η_3*i*_ = *s*_1*i*_Η_1*i*_, (Equation 64) can be rewritten as

(Equation 71) $${\dot{V}}_{i}\left(s_{1i},s_{2i}\right)\leq -\left(-{\bar{h}}_{Mi}+L_{bi}\left(s_{1i}\right)\right)\left|s_{1i}\right|-K_{3i}\left|s_{2i}\right|+\frac{1}{2}{\bar{\varphi }}_{i}^{2}$$

By the definition of the auxiliary variable *ϕ*_*i*_, for any 0<*ϕ*_*i*_<|*s*_1*i*_|<*κ*_*i*_, it always holds that

(Equation 72) $${\bar{h}}_{Mi}=L_{bi}\left(\phi_{i}\right)<L_{bi}\left(s_{1i}\right)$$

Therefore, we can conclude that

(Equation 73) $${\dot{V}}_{i}\left(s_{1i},s_{2i}\right)\leq -\Theta_{i}V_{i}^{\frac{1}{2}}\left(s_{1i},s_{2i}\right)+\frac{1}{2}{\bar{\varphi }}_{i}^{2}$$

where $\Theta_{i}=\sqrt{2}\min\left\{-{\bar{h}}_{Mi}+L_{bi}\left(s_{1i}\right),K_{3i}\right\},i=1,2,3$. Then, from Equation 72 and Lemma 4, it follows that

(Equation 74) $$\dot{V}\left(s_{1},s_{2}\right)\leq -\Theta V^{\frac{1}{2}}\left(s_{1},s_{2}\right)+Ζ$$

where Θ = *min*{Θ_1_,Θ_2_,Θ_3_} and $Ζ=\frac{1}{2}\sum_{i=1}^{3}{\bar{\varphi }}_{i}^{2}$.

By applying Lemma 3 and (Equation 74), the system will achieve finite-time stability, with the convergence time governed by

(Equation 75) $$T\leq \frac{2}{\varpi \Theta }V^{\frac{1}{2}}\left(s_{1}\left(0\right),s_{2}\left(0\right)\right)$$

where 0<*ϖ* < 1; *V*(*s*_1_(0),*s*_2_(0)) is the initial value of *V*(*s*_1_,*s*_2_).

Thus, under the proposed control strategy, the sliding mode variable *s*_1*i*_ converges to the interval [-*κ*_*i*_,*κ*_*i*_] in finite time for *ϕ*_*i*_<|*s*_1*i*_|<*κ*_*i*_. Using Equations 29 and 13, it follows that

(Equation 76) $${\dot{x}}_{1i}=-\rho_{i}x_{1i}+s_{1i}+{\tilde{d}}_{1i}+e^{-\alpha_{i}t}{\bar{s}}_{i}\left(0\right)$$

Based on |*s*_1*i*_|≤*κ*_*i*_ and $\left|{\tilde{d}}_{1i}\right|\leq \varepsilon_{1}$, one yields

(Equation 77) $$\left|x_{1i}\right|=\left|x_{1i}\left(0\right)e^{-\rho_{i}t}+\int_{0}^{t}\left(s_{1i}\left(\chi \right)+{\tilde{d}}_{1i}\left(\chi \right)+{\bar{s}}_{1i}\left(0\right)e^{-\alpha_{i}\chi }\right)e^{-\rho_{i}\left(t-\chi \right)}d\chi \right|\leq \left|x_{1i}\left(0\right)\right|e^{-\rho_{i}t}+\frac{\kappa_{i}+\varepsilon_{1}}{\rho_{i}}\left(1-e^{-\rho_{i}t}\right)+\frac{{\bar{s}}_{1i}\left(0\right)e^{-\rho_{i}\chi }}{\alpha_{i}-\rho_{i}}\left(1-e^{-\left(\alpha_{i}-\rho_{i}\right)t}\right)$$

Since *ρ*_*i*_ > 0, and in view of Equation 77, the following result is derived:

(Equation 78) $$\left|x_{1i}\right|\leq \frac{\kappa_{i}+\varepsilon_{1}}{\rho_{i}},t\to \infty$$

Similarly, when *t*→*∞*, the following holds

(Equation 79) $$\left|x_{2i}\right|=\rho_{i}\left|x_{1i}\right|+\left|s_{1i}\right|+\left|d_{1i}\right|+\left|{\tilde{d}}_{1i}\right|+\Pi_{i}\left|{\bar{s}}_{1i}\left(0\right)\right|\leq 2\kappa_{i}+2\varepsilon_{1}+\mu_{1i}$$

In the light of Equations 78 and 79, it can be inferred that, under the improved SMC strategy, the system state variables *x*_1*i*_ and *x*_2*i*_ will converge to the designated regions |*x*_1*i*_|≤(*κ*_*i*_+*ε*_1_)/*ρ*_*i*_ and |*x*_2*i*_|≤2*κ*_*i*_+2*ε*_1_+*μ*_1*i*_,*i* = 1,2,3 within a finite time, respectively. Given that *x*_1_ = *e*, the improved SMC strategy ensures that the trajectory tracking error remains within a neighborhood of the origin while preventing overestimation of the control gains. Furthermore, the convergence neighborhood can be adjusted by appropriately selecting the control gains *ρ*_1_,*α*_1_,*L*_1_,*L*_2_,*K*_0_,*K*_1_,*K*_2_,*K*_3_ and *κ*_*i*_,*i* = 1,2,3. This completes the proof of Theorem 3.

Remark 5. To ensure the continuity of the control signal, a continuous approximation function *s*_2*i*_/(‖*s*_2_‖+*l*_*i*_),*i* = 1,2,3 can be employed to replace the sign function *sign*(*s*_2*i*_) in the auxiliary control signal (59), where *l*_*i*_ is a small positive constant, thereby mitigating system chattering. In contrast, the term *L*_*bi*_(*s*_1*i*_)*sign*(*s*_1*i*_) in the intermediate control vector (51) requires no modification, as $L_{bi}\left(s_{1i}\right)\text{sgn}\left(s_{1i}\right)=\frac{s_{1i}}{\kappa_{i}-\left|s_{1i}\right|}$ is inherently continuous.

Remark 6. The improved SMC strategy ensures that the sliding mode surface *s*_1*i*_ converges to a neighborhood of the origin from any initial condition *s*_1*i*_(0). As *s*_1*i*_ approaches the boundary of the interval [-*κ*_*i*_,*κ*_*i*_], the value of the PSDB function increases, generating sufficient control force to steer *s*_1*i*_ back into neighborhood [-*κ*_*i*_,*κ*_*i*_]. This mechanism ensures both the stability of the sliding mode surface and the overall closed-loop system.

### Numerical simulations

To validate the effectiveness of proposed SMC strategies, numerical simulations are performed for trajectory tracking of the fully-actuated USV Cybership-II under various scenarios.^35^^,^^36^ The mathematical model parameters for the USV are provided as *M* = [*m*_11_,0,0;0,*m*_22_,*m*_23_;0,*m*_32_,*m*_33_], *C*(*v*) = [0,0,*c*_13_;0,0,*c*_23_;-*c*_13_,-*c*_23_,0], and *D*(*v*) = [*d*_11_,0,0;0,*d*_22_,*d*_23_;0,*d*_32_,*d*_33_], where *m*_11_ = 25.8, *m*_22_ = 33.8, *m*_23_ = *m*_32_ = 1.0948, *m*_33_ = 2.76, *c*_13_ = −33.8*υ*-1.0948*r*, *c*_23_ = 25.8*u*, *d*_11_ = 0.7225 + 1.3274|*u*|+5.8664*u*^2^, *d*_22_ = 0.8612 + 36.2823|*υ*|+0.805|*r*|, *d*_33_ = 1.9–0.08|*υ*|+0.75|*r*|, *d*_23_ = −0.1079 + 0.845|*υ*|+3.45|*r*|, and *d*_32_ = −0.1052–5.0437|*υ*|-0.13|*r*|. The input saturation amplitudes are set as *τ*_*M*1_ = 5[N], *τ*_*M*2_ = 5[N], and *τ*_*M*3_ = 3.5[Nm], respectively.^37^

The reference trajectory is set as

(Equation 80) $$\eta_{d}=\left\{ \begin{array}{l} \left[0.5t;0;0.01t\right],if\;t\leq 40\\ \left[20+50\;\sin\left(t^{\prime }\right);50\left(1-\sin\left(t^{\prime }\right)\right);0.01t\right],if\;t>40 \end{array}\right.$$

where *t*′ = 0.01*t*-40.

Table 1 summarizes the chosen control design parameters, while the initial conditions for the simulation are set as *η*_0_ = [2,2,*π*/4]^*T*^, *v*(0) = [0,0,0]^*T*^ and *ς*(0) = [1,1,1]^*T*^.

**Table 1:** The control design parameters

Strategies	Parameters
Composite disturbance observer	*L*_1_ = *diag*([0.25,0.25,0.25]), *L*_2_ = *diag*([1,1,1])
SMC strategy	*ρ* = *diag*([0.1,1,1]), *α* = *diag*([0.02,0.1,0.1]), *K*_0_ = *diag*([5,5,5]), *K*_1_ = *diag*([0.8,2.5,2.5]), *K*_2_ = 10^−3^×*diag*([1,1,1])
Improved SMC strategy	*ρ* = *diag*([0.1,1,1]), *α* = *diag*([0.02,0.1,0.1]), *K*_1_ = *diag*([0.8,2.5,2.5]), *K*_2_ = *diag*([0.1,0.1,0.1]), *K*_3_ = 10^−4^×*diag*([3,2,5]), *T*_1_ = 0.01, *l*_*i*_ = 10^−4^, *κ*_*i*_ = 0.08, *ζ* = 10^−3^×*diag*([2,2,2]), $\bar{V}=diag\left(\left[0.3,0.3,0.3\right]\right)$

### Scenario 1

In this scenario, simulation results are contrasted with those of an event-triggered adaptive trajectory tracking strategy *τ*_*a*_ for the USV, demonstrating the advantages of the proposed strategies. While the event-triggered adaptive trajectory tracking strategy mitigates unknown matched disturbances, it fails to adequately address the issues of unmatched disturbances and input saturation constraints. The formulation of *τ*_*a*_ and the selection of its design parameters are informed by Gao et al.^37^

The unmatched disturbance is chosen as

(Equation 81) $$d_{u}\left(t\right)=0.1\times \left[\begin{array}{l} 3.5u^{3}-0.5u^{2}\;\sin\left(2t\right)\\ 3.5v^{3}-0.5v^{2}\;\sin\left(2t\right)\\ 3.5r^{3}-0.5r^{2}\;\sin\left(2t\right) \end{array}\right]$$

The matched disturbance is chosen as

(Equation 82) $$d_{m}\left(t\right)=\left[\begin{array}{l} 2.3+\sin\left(0.02t\right)+1.5\;\sin\left(0.01t\right)\\ -2.9+\sin\left(0.02t-\pi /6\right)+1.5\;\sin\left(0.03t\right)\\ -\sin\left(0.09t+\pi /3\right)-\sin\left(0.2t\right) \end{array}\right]$$

**Figure 4:**
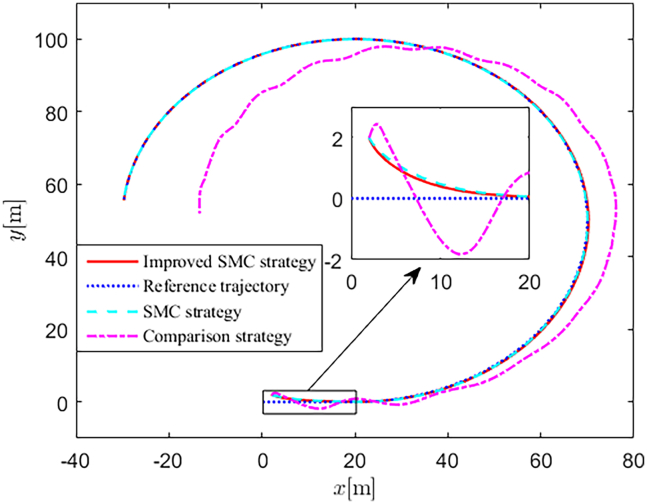
Trajectory tracking in scenario 1

**Figure 5:**
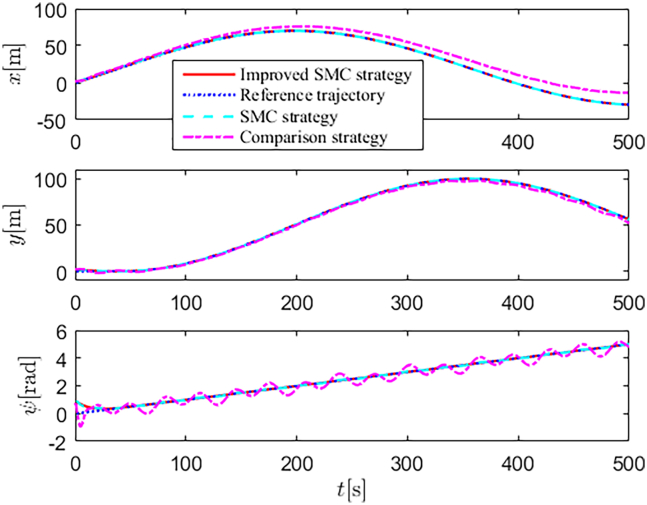
Trajectory tracking in different directions in scenario 1

**Figure 6:**
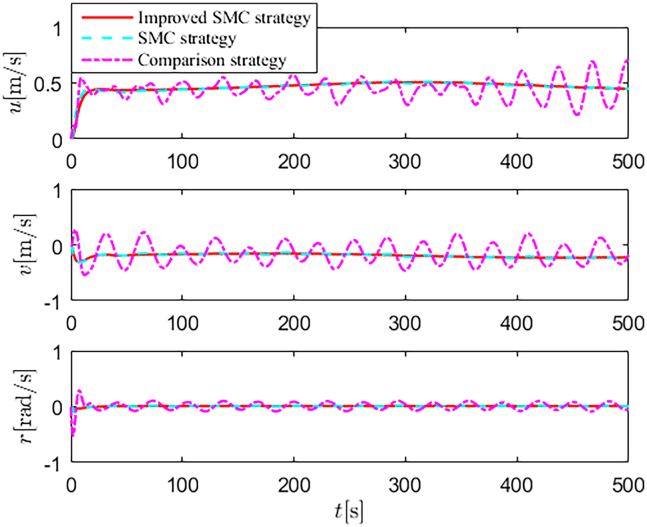
Velocity profiles in scenario 1

**Figure 7:**
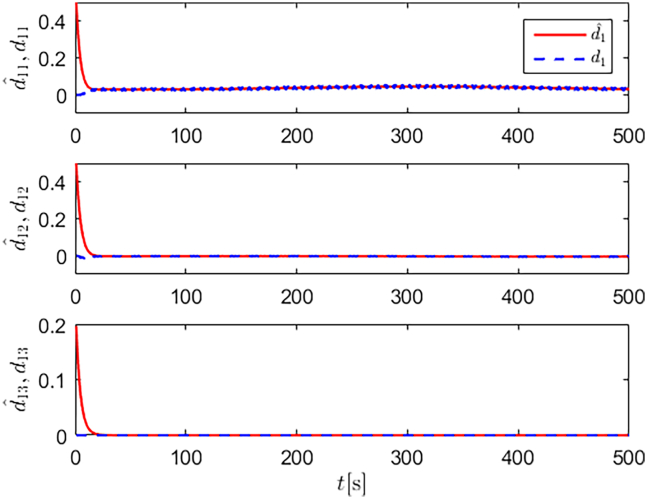
Unmatched disturbances and their estimates under the improved SMC strategy in scenario 1

**Figure 8:**
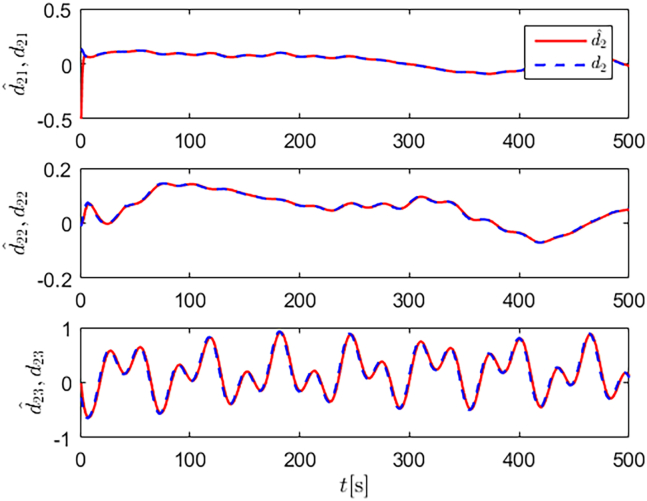
Matched disturbances and their estimates under the improved SMC strategy in scenario 1

**Figure 9:**
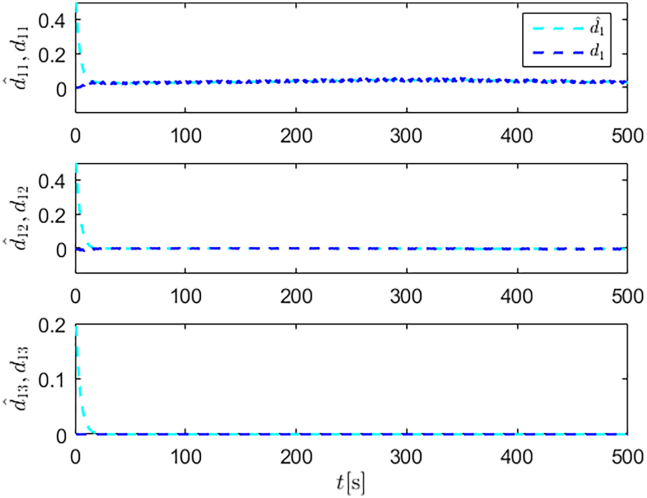
Unmatched disturbances and their estimates under the SMC strategy in scenario 1

**Figure 10:**
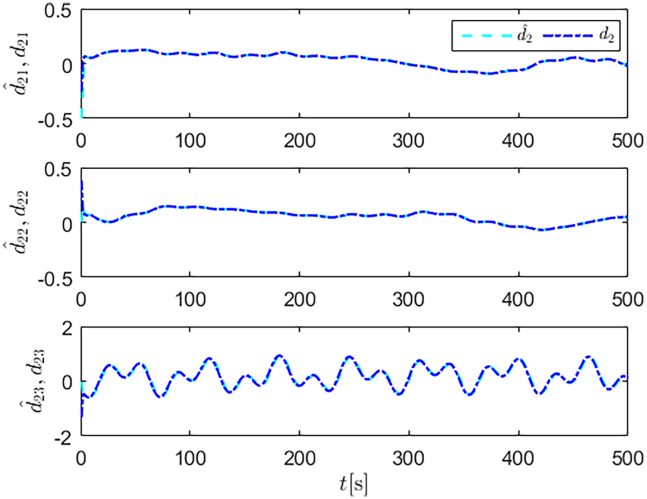
Matched disturbances and their estimates under the SMC strategy in scenario 1

**Figure 11:**
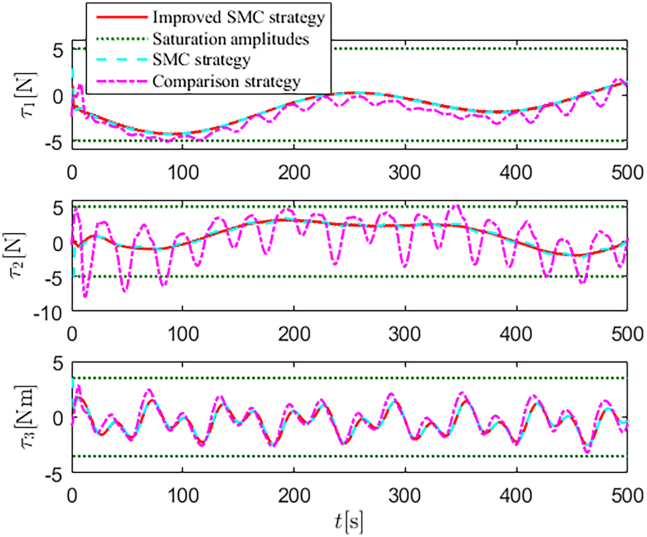
Control inputs in scenario 1

**Figure 12:**
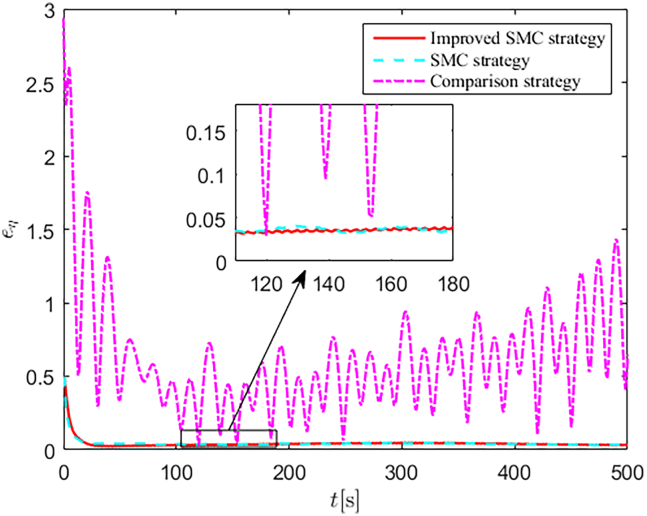
Control performances in scenario 1

Simulation results are shown in Figures 4, 5, 6, 7, 8, 9, 10, 11, and 12. Figures 4 and 5 demonstrate that, under the proposed strategies, the USV accurately tracks the reference trajectory, while the comparison algorithm *τ*_*a*_ lacks precise tracking control capabilities. Figure 6 presents the velocity curves; under the proposed strategies, the velocities remain smooth and bounded. As depicted in Figures 7, 8, 9, and 10, the composite disturbance observer provides rapid response and excellent approximation performance in estimating and compensating for matched and unmatched disturbances. These results confirm that the designed disturbance observer effectively suppresses the influence of composite disturbances on the system. Figure 11 illustrates the control inputs. Notably, the control input of the comparison algorithm violates the saturation constraints, whereas the proposed algorithms maintain the control signals strictly within the saturation bounds. Figure 12 presents the control performance $e_{\eta }=\sqrt{e_{1}^{2}+e_{2}^{2}+e_{3}^{2}}$, further corroborating the superiority of the proposed algorithms. In summary, in this scenario, both the SMC strategy and the improved SMC strategy both yield effective trajectory tracking performance.

### Scenario 2

The unmatched disturbance is chosen as

(Equation 83) $$d_{u}\left(t\right)=0.1\times \left[\begin{array}{l} 7u^{3}-u^{2}\;\sin\left(2t\right)\\ 7v^{3}-v^{2}\;\sin\left(2t\right)\\ 7r^{3}-r^{2}\;\sin\left(2t\right) \end{array}\right]$$

Unlike scenario 1, the matched disturbance in this scenario is derived from the first-order Markov process:

(Equation 84) $$d_{m}\left(t\right)=J^{T}\left(\psi \right)\hbar$$

(Equation 85) $$\dot{\hbar }=-\zeta^{-1}\hbar +\bar{V}\Lambda$$

where $\hbar \in R^{3\times 1}$ represents the disturbance vector from the first-order Markov process; $\zeta \in R^{3\times 3}$ is the time constant matrix; $\Lambda \in R^{3\times 1}$ denotes zero-mean Gaussian white noise; $\bar{V}\in R^{3\times 3}$ is the amplitude matrix of Λ.

**Figure 13:**
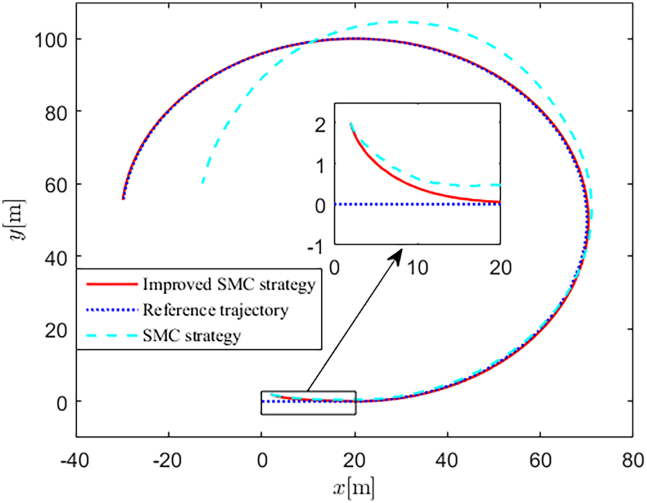
Trajectory tracking in scenario 2

**Figure 14:**
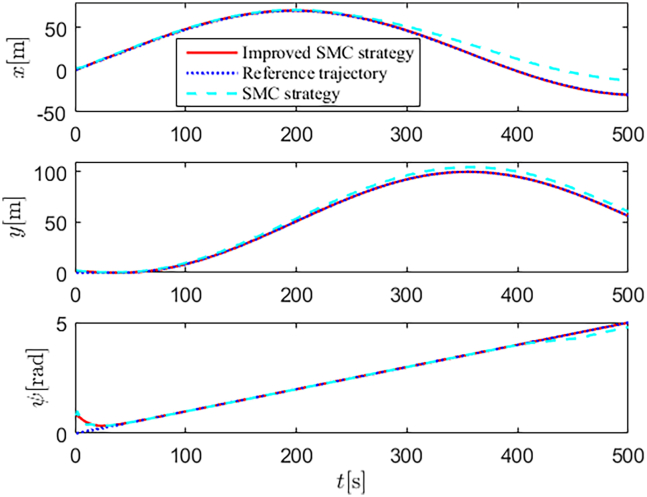
Trajectory tracking in different directions in scenario 2

**Figure 15:**
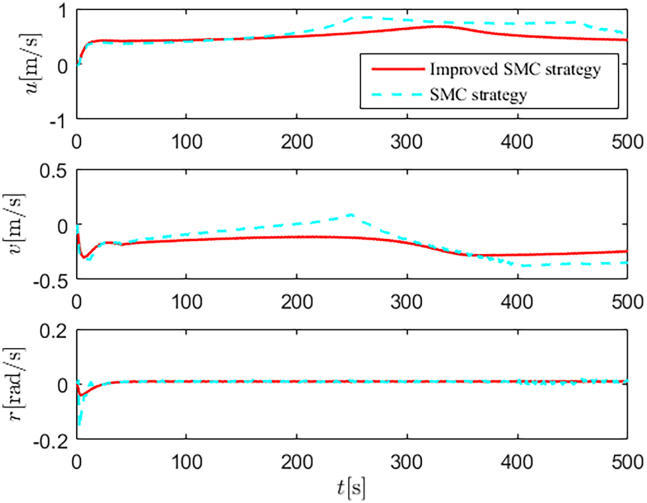
Velocity profiles in scenario 2

**Figure 16:**
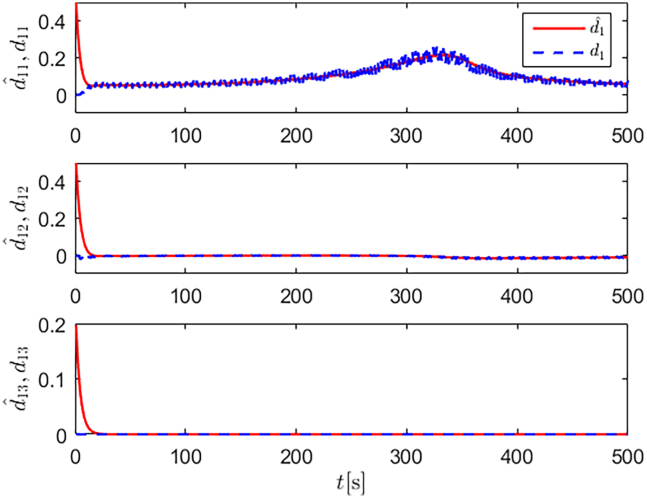
Unmatched disturbances and their estimates under the improved SMC strategy in scenario 2

**Figure 17:**
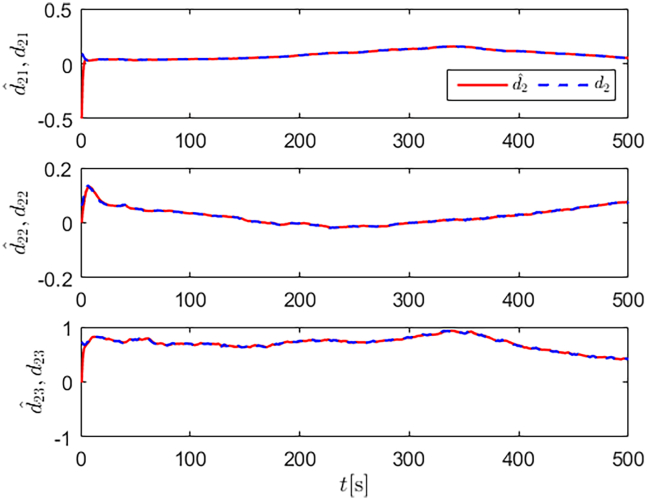
Matched disturbances and their estimates under the improved SMC strategy in scenario 2

**Figure 18:**
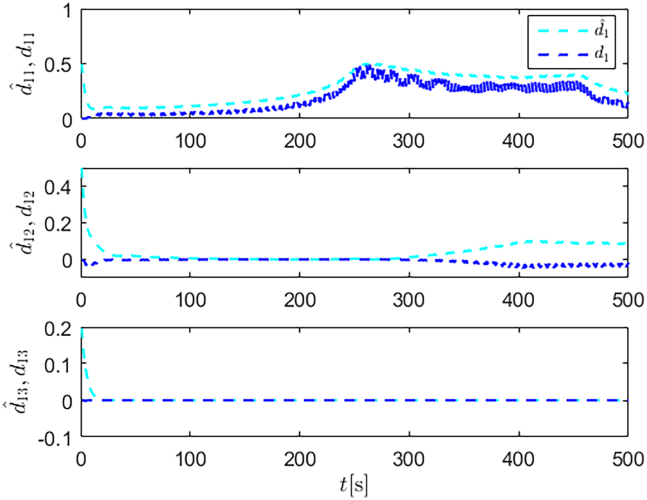
Unmatched disturbances and their estimates under the SMC strategy in scenario 2

**Figure 19:**
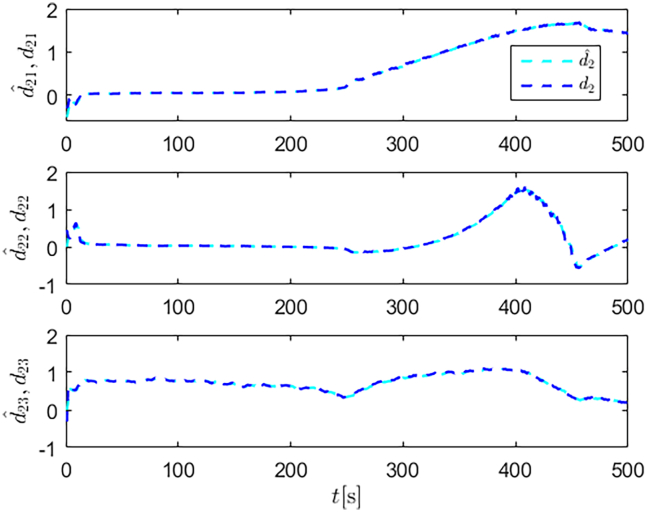
Matched disturbances and their estimates under the SMC strategy in scenario 2

**Figure 20:**
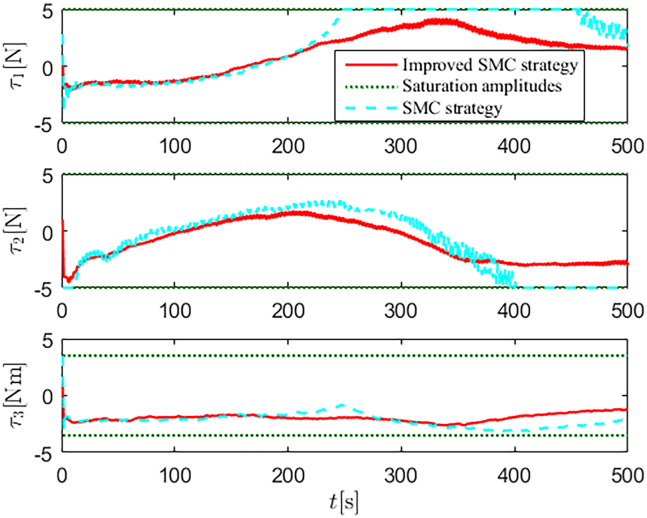
Control inputs in scenario 2

**Figure 21:**
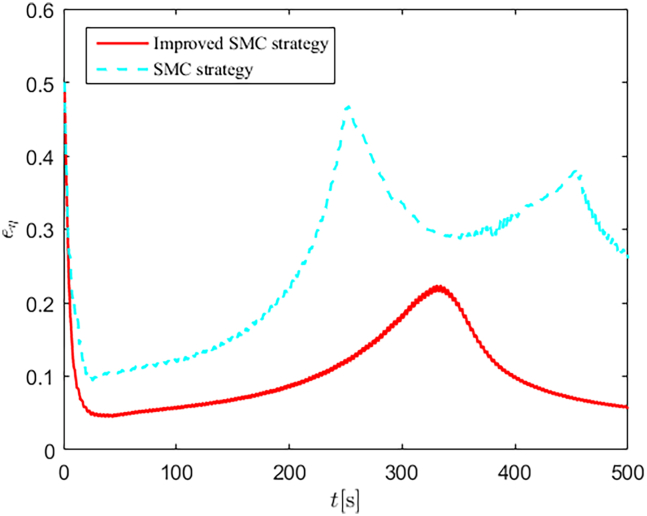
Control performances in scenario 2

The selection of parameters is provided in Table 1, and the simulation initial condition is set as ℏ(0) = [2,2,2]^*T*^. Simulation results are shown in Figures 13, 14, 15, 16, 17, 18, 19, 20, and 21. It is evident that the improved SMC strategy maintains system control performance even in the presence of large unmatched disturbances. In contrast, the proposed SMC strategy fails to effectively handle large disturbances, resulting in significant degradation in trajectory tracking accuracy. This performance degradation may arise from the selected the control gain *K*_2_ failing to meet the condition (6), resulting in an overestimation of the control gains and a consequent significant reduction in system performance.

**Figure 22:**
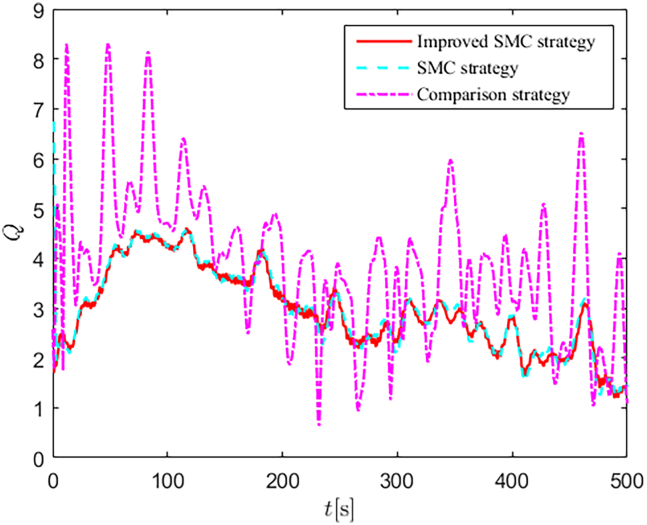
Energy consumption in scenario 1

**Figure 23:**
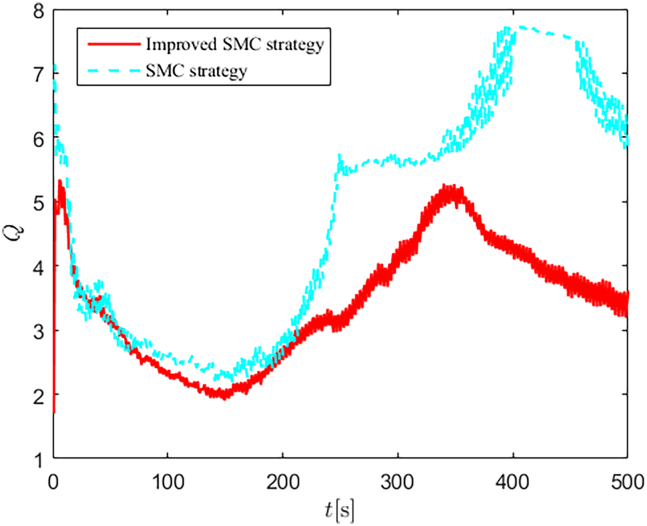
Energy consumption in scenario 2

Figures 22 and 23 depict the energy consumption $Q=\sqrt{\tau_{1}^{2}+\tau_{2}^{2}+\tau_{3}^{2}}$ response curves for both scenarios. In scenario 1, the proposed control strategies efficiently estimate and compensate for unmatched and matched disturbances, thereby achieving lower energy consumption compared to the comparison algorithm. In scenario 2, the proposed SMC strategy failed to achieve satisfactory control performance, resulting in higher energy consumption compared to the improved SMC strategy. This further validates the superiority of the improved SMC strategy, particularly in terms of effective disturbance suppression and strong non-fragility.

## Discussion

This study presents two SMC strategies for trajectory tracking, tailored for a fully actuated USV facing unmatched and matched disturbances as well as input saturation constraints. Initially, a composite disturbance observer is developed to estimate both unmatched and matched disturbances. Subsequently, an SMC method is proposed that integrates an auxiliary dynamic system to achieve finite-time convergence of tracking errors. An improved SMC strategy is further introduced, featuring a PSDB function and a saturation approximation model. This enhancement guarantees system state convergence within a finite time, avoids overestimating control gains, and ensures smooth control inputs. Simulation results validate the proposed strategies’ efficacy and highlight their superiority over existing methods.

### Limitations of the study

The current study does not impose constraints on the system’s dynamic performance, which limits the practical application of the proposed control strategies. This issue can be addressed in future work by incorporating prescribed performance control techniques.

## Resource availability

### Lead contact

Requests for further information and resources should be directed to and will be fulfilled by the lead contact, Dr. Chenlong Gong (gongcl@whut.edu.cn).

### Materials availability

This study did not generate new unique reagents.

### Data and code availability

• All data reported in this paper will be shared by the lead contact upon reasonable request.
• All code in this paper will be shared by the lead contact upon reasonable request.
• Any additional information required to reanalyze the data reported in this paper is available from the lead contact upon reasonable request.

## Acknowledgments

This work is supported by the Fundamental Research Funds for the Central Universities (104972024RSCbs0038).

## Author contributions

Validation, Y.S. and C.G.; written, Y.S., C.G., and D.Z.; checked, Y.S. and C.G.; conceptualization, Z.L. and Y.S.; methodology, Z.L. and D.Z.; software. Z.L. and C.G.

## Declaration of interests

The authors declare no conflict of interest.

## Declaration of generative AI and AI-assisted technologies in the writing process

During the preparation of this work, the author(s) used ChatGPT in order to polish the language. After using this tool or service, the author(s) reviewed and edited the content as needed and take(s) full responsibility for the content of the publication.

## STAR★Methods

### Experimental model and study participant details

Omitted as our study does not involve biological models.

### Method details

This study validates the proposed algorithm through numerical simulations conducted in MATLAB (R2015b). The USV model adopted is CYBERSHIP-II, and its detailed parameters are taken from the studies by Skjetne et al.^35^ and Su et al.^36^ The input saturation limits are selected according to the work of Gao et al.^37^

### Quantification and statistical analysis

No statistical methods, sample sizes, and software was used/generated in the study.
